# The role of inhibition in the generation of reliable spike sequences

**DOI:** 10.1186/1471-2202-14-S1-P11

**Published:** 2013-07-08

**Authors:** Collins Assisi

**Affiliations:** 1 Indian Institute of Science Education and Research, Pune, India, 411007

## 

Coordinated spiking of neuronal populations underlies the perception of sensory stimuli, the planning of movement, and the acquisition of new memories. In the hippocampus and the entorhinal cortex, groups of neurons are transiently activated as an animal traverses its environment. The sequential patterns generated by these neurons depend, not only on environmental cues, but can also be generated reliably in the absence of these cues. Such patterning depends on the temporal and the spatial distribution of inhibition. It has been hypothesized that inhibitory interneurons corral principal neurons into transiently synchronous ensembles that encode sensory information and sub-serve behavior. The goal of this study is to demonstrate a link between the topology of the inhibitory sub-network and the dynamics of its constituent neurons that, in turn, support the generation of reliable sequences of spiking activity.

An understanding of structure-dynamics relationships in complex networks is often challenging because of the formidable dimensionality of the system and the inherent non-linear properties of neurons and synapses that constitute the network. In an earlier study [1] we established a relationship between a structural property of the network, its colorings (a proper vertex coloring of a network is one that assigns different colors to nodes that are directly connected to each other), and the dynamics it constrains. Competitive inhibitory interactions ensure that neurons associated with the same color generate spikes in approximate synchrony. Further we showed that the description of network dynamics based on its coloring comes with a fortuitous benefit. We used the coloring of the inhibitory sub-network as a tool to construct a space in which the distance between excitatory neurons is defined not by the length of the synaptic path connecting those neurons but by the similarity of the inhibitory input they receive. In this reconfigured space, the seemingly high-dimensional dynamics of networks of randomly connected excitatory and inhibitory neurons reliably forms a series of orthogonally propagating waves, a predictable and simple pattern where synchronous ensembles of excitatory neurons are successively recruited.

The coloring constrains the identity of inhibitory neurons that spike synchronously. However, it does not impose any constraints on the order in which synchronous groups are recruited. The propensity of a particular group of neurons to spike can be attributed to slow temporal processes such as adaptive changes in the calcium concentration within the cell and slow oscillatory drive over time scales comparable to that of theta oscillations that are observed in the hippocampus and the entorhinal cortex. In this study we systematically vary the topology of the network (exemplified by it's coloring) and the timescale of slow temporal processes to understand how the two interact to construct reliable sequences of activity.
